# Supplementary material for: Transcriptional targets of senataxin and E2 promoter binding factors are associated with neuro-degenerative pathways during increased autophagic flux
Source: Sci Rep. 2022 Oct 21;12:17665. doi: 10.1038/s41598-022-21617-2 (PMC9587291; doi:10.1038/s41598-022-21617-2)
Supplement: Supplementary file 1 — Supplementary Information 1. [file 41598_2022_21617_MOESM1_ESM.pdf]

Transcriptional targets of senataxin and E2 promoter binding factors are associated with neuro-degenerative pathways during increased autophagic flux

Supplementary files

Aaron E. Casey[1,2,#,*], Wenjun Liu[3,#], Leanne K. Hein[4], Timothy J. Sargeant[4], Stephen M. Pederson[3], Ville-Petteri Mäkinen[1,2,5,6]

[1]Computational and Systems Biology Program, Precision Medicine Theme, South Australian Health and Medical Research Institute, Adelaide, Australia
[2]Australian Centre for Precision Health, Cancer Research Institute, University of South Australia, Adelaide, Australia
[3]Dame Roma Mitchell Cancer Research Laboratories, Adelaide Medical School, Faculty of Health and Medical Sciences, University of Adelaide, Adelaide, Australia
[4]Lysosomal Health in Ageing, Hopwood Centre for Neurobiology, Lifelong Health Theme, South Australian Health and Medical Research Institute, Adelaide, Australia
[5]Computational Medicine, Faculty of Medicine, University of Oulu, Oulu, Finland
[6]Center for Life Course Health Research, Faculty of Medicine, University of Oulu, Oulu, Finland

[#]Equal contribution

*Corresponding author
Aaron Casey
Address: SAHMRI North Terrace, Adelaide, SA 5000, Australia
Email: aaron.casey@sahmri.com

A

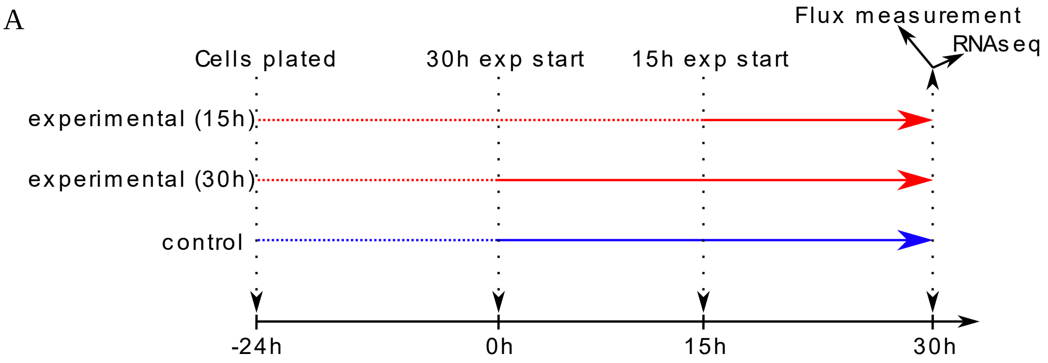

B

Cell Line	Experiment	Condition	Intervention	Time
HEK293	mTOR inhibition	experimental	AZD8055	15h
				30h
		control	DMEM	30h
	Starvation	experimental	EBSS	15h
				30h
		control	DMEM	30h
HeLa	mTOR inhibition	experimental	AZD8055	15h
				30h
		control	DMEM	30h
	Starvation	experimental	EBSS	15h
				30h
		control	DMEM	30h
SH-SY5Y	mTOR inhibition	experimental	AZD8055	15h
				30h
		control	DMEM	30h
	Starvation	experimental	EBSS	15h
				30h
		control	DMEM	30h

Supplementary figure S1. (A) Schematic illustration of experimental protocol repeated for each cell line (HEK 293, HeLa and SH-SY5Y) and condition (mTOR inhibition and starvation). Inhibition of the mTOR complexes was achieved by the chemical compound AZD8055. Starvation was induced by applying Earle's Balanced Salt Solution (EBSS) as culture media. (B) Outline of all final experiments carried out for DE gene analysis.

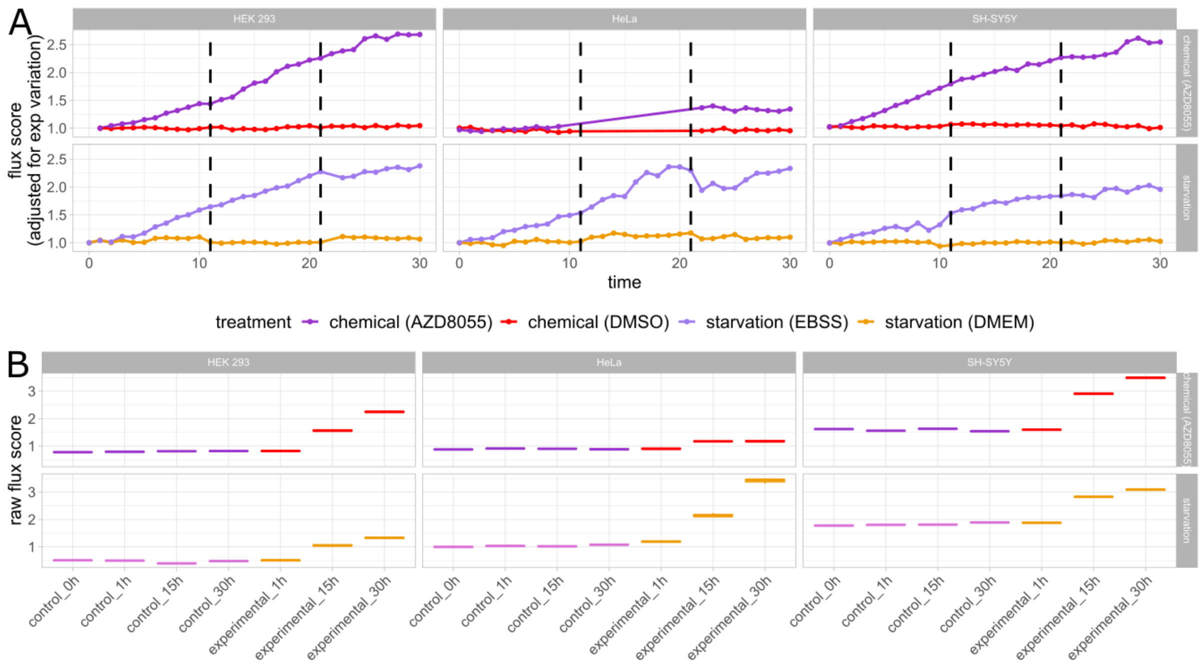

Supplementary figure S2. Assessment of autophagic flux using the tf-LC3 assay. The flux score was defined as the ratio between the red and green fluorophores (see Methods). **A**) Monoclonal cultures of three cell lines that were subjected to starvation (EBSS) and chemical mTOR inhibition (AZD8055). Samples were obtained every hour to determine the time curves of autophagy responses. **B**) Measurements of autophagic flux from the samples that were sent to RNA-sequencing. Interquartile box plots of three replicates are shown, although variation was so low that the boxes got flattened for most time points.

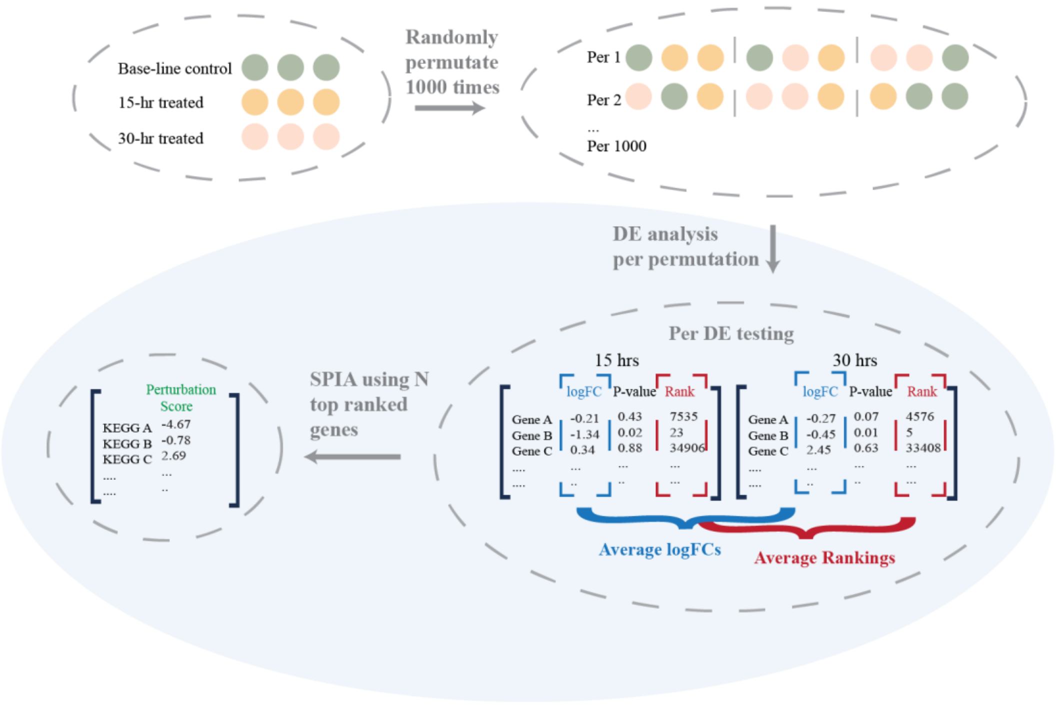

Supplementary Figure S3. SPIA

In Signaling Pathway Impact Analysis (SPIA), the probability of obtaining the observed perturbation score (PS) is calculated through a bootstrap approach, where random genes in the same numbers as DE genes inputted would be assigned to random locations of pathways and receive logFCs randomly sampled from the range of logFC as observed within DE genes. The underlying assumption of this approach is that genes are statistically independent, while the primary purpose of gene-sets was to group co-regulated genes, thus challenging the assumption. Moreover, assigning genes randomly breaks the gene-gene correlations, which could result in over-estimated statistical significance [Dørum, G., et al., Rotation testing in gene set enrichment analysis for small direct comparison experiments. Stat Appl Genet Mol Biol, 2009. 8: p. Article34. Efron, B. and R. Tibshirani, On testing the significance of sets of genes. The annals of applied statistics, 2007. 1(1): p. 107-129.]. Therefore, we developed an alternative significance testing method that permutes samples instead to simulate null distributions of KEGG pathways PSs.

To simulate null distributions of PSs, we firstly generated permuted logFCs by shuffling the 9 samples within each condition (3 base-line control, 3 15-h treated and 3 30-h treated) and assigning the first 3 samples of each permutation to be base-line controls, middle 3 to be 15-h treated and last 3 to be 30-h treated samples. 1000 permutations were randomly sampled from the total 362,880 possible permutations and differential expression analyses were performed 1000 times following methods described in the ‘Differential Expression Analysis” section. To reduce computation time, dispersion

estimation required in the edgeR workflow was only performed once, assuming that all samples were derived from the same group.

For each round of DE testing, genes were ranked by p-values at both time points, and the final rankings were derived through ranking the mean of two individual rankings. The same number of top-ranked genes as the number of DEGs defined under each condition were kept and used as the permuted DEGs. Again, average logFCs between 15 h and 30 h were taken for each DE testing to derive a single estimate of FCs, thus giving rise to 1000 sets of permuted DEGs with permuted logFCs for each condition.

SPIA's net perturbation accumulation algorithm was then applied 1000 times utilising the permutated DEGs and their logFCs to derive the null distribution of perturbation scores for each KEGG pathway, from which the mean standard deviation (MAD) of each pathway was calculated. As the default of mad function in the *stats* package, the actual value outputted was constant × MAD, where the constant was defaulted to be 1.4826 to approximate standard deviation. Statistical significance of observed PS was then defined by firstly calculating the corresponding robust z-scores:$\frac{(tA-median)}{scaled\ MAD}$. The robust z-scores were then converted to 2-sided p-values and multiple testing consideration was accounted for through the Benjamini-Hochberg method. KEGG pathways with perturbation FDR smaller than 0.05 were deemed to be significantly perturbed.

Supplementary Figures S4 – S8: Genes discussed in manuscript that were significantly differentially expressed in the same direction for all cell lines in both mTOR inhibition and starvation experiments

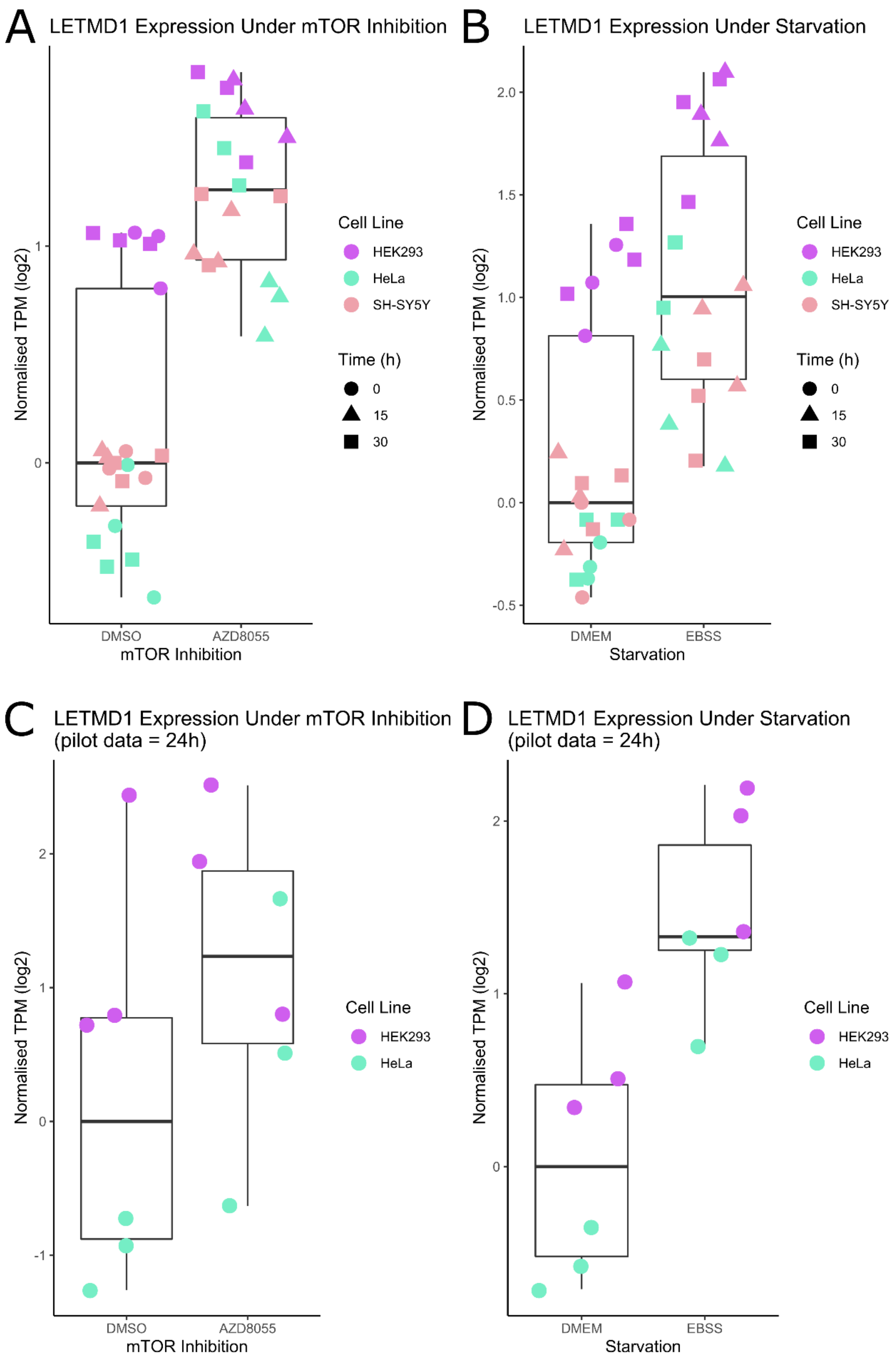

Supplementary Figure S4. Normalised LETMD1 expression as transcripts per million (TPM) in primary experiments (A and B) and pilot experiments (C and D). mTOR inhibition and starvation experiments are represented in (A and C) and (B and D) respectively. Pilot experiments lacked SH-SY5Y data and were exposed to experimental stimulus for 24 hours.

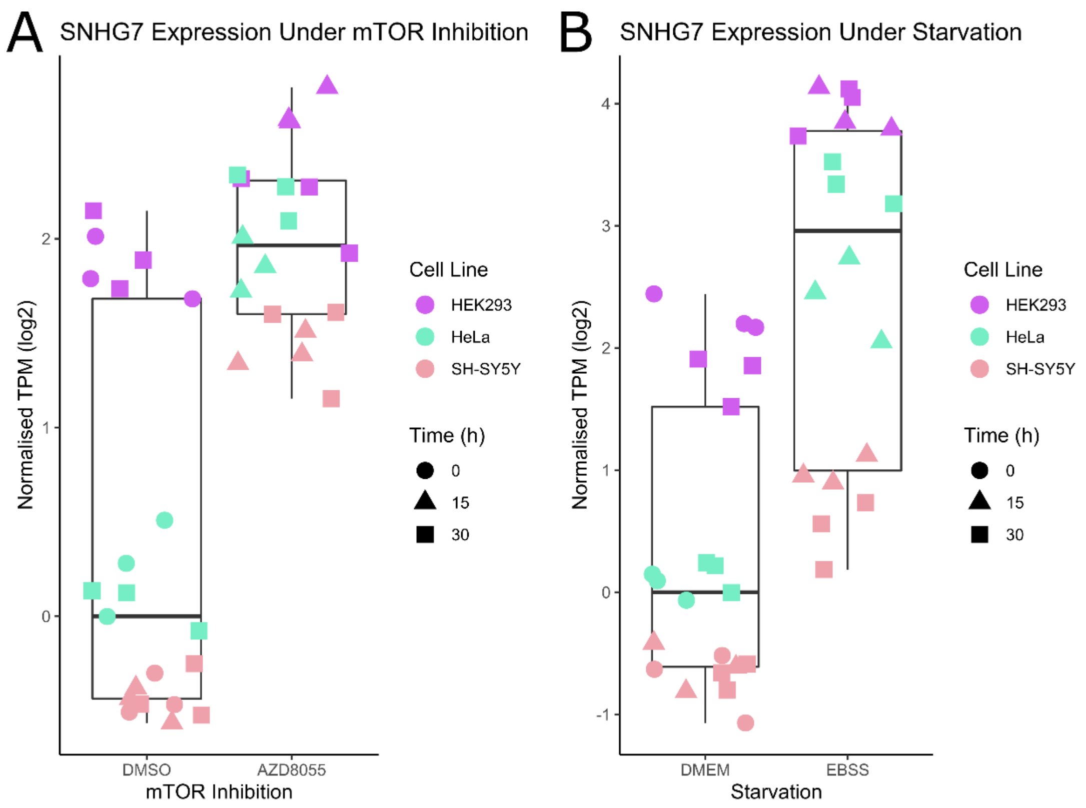

C

non-coding RNA
not captured in
pilot data

D

non-coding RNA
not captured in
pilot data

Supplementary Figure S5. Normalised SNHG7 expression as transcripts per million (TPM) in primary experiments (A and B) and pilot experiments (C and D). mTOR inhibition and starvation experiments are represented in (A and C) and (B and D) respectively. Pilot experiments lacked SH-SY5Y data and were exposed to experimental stimulus for 24 hours. Pilot data also did not capture non-coding RNA, thus there is no data for SNHG7.

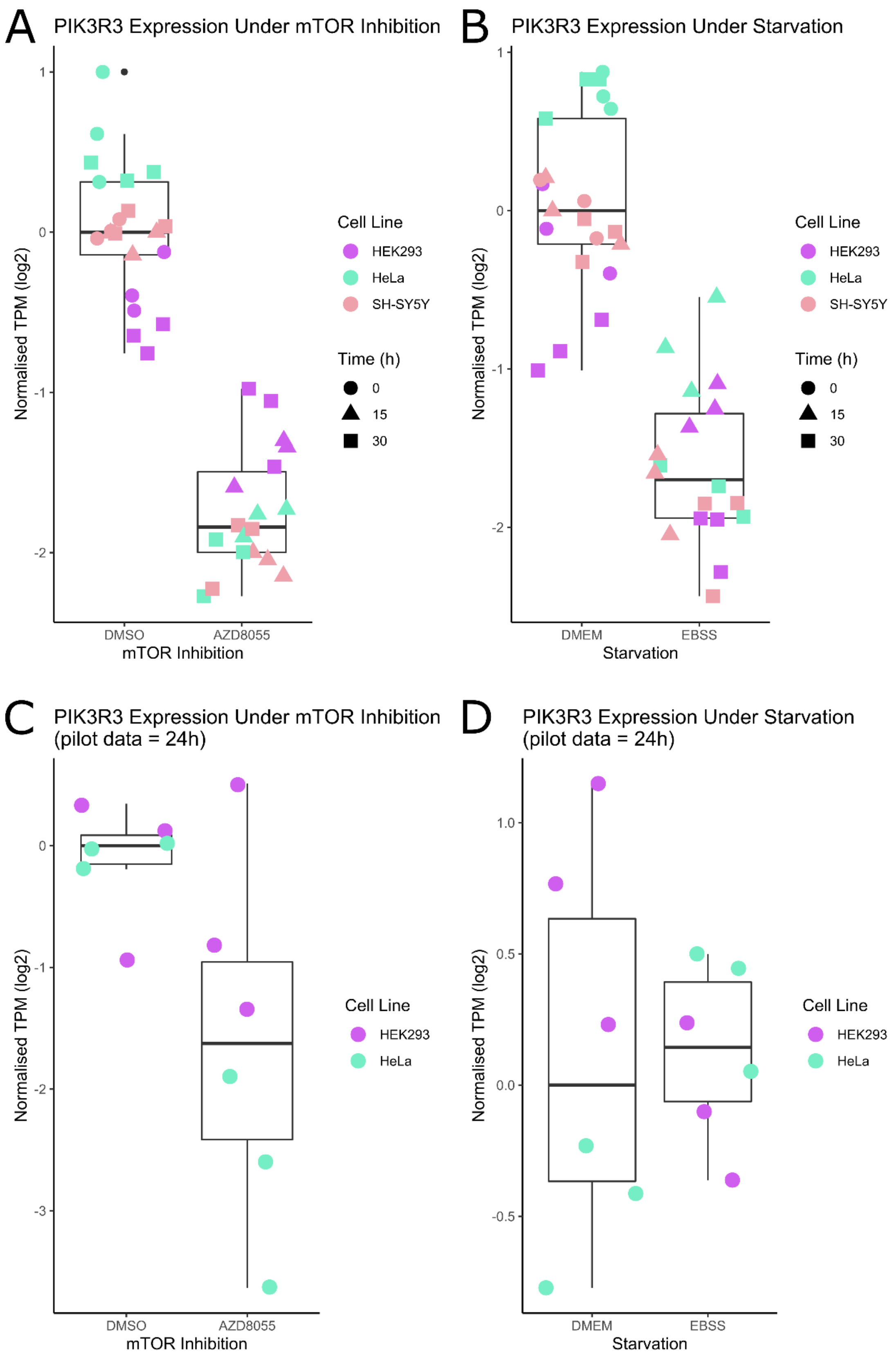

Supplementary Figure S6. Normalised PIK3R3 expression as transcripts per million (TPM) in primary experiments (A and B) and pilot experiments (C and D). mTOR inhibition and starvation experiments are represented in (A and C) and (B and D) respectively. Pilot experiments lacked SH-SY5Y data and were exposed to experimental stimulus for 24 hours.

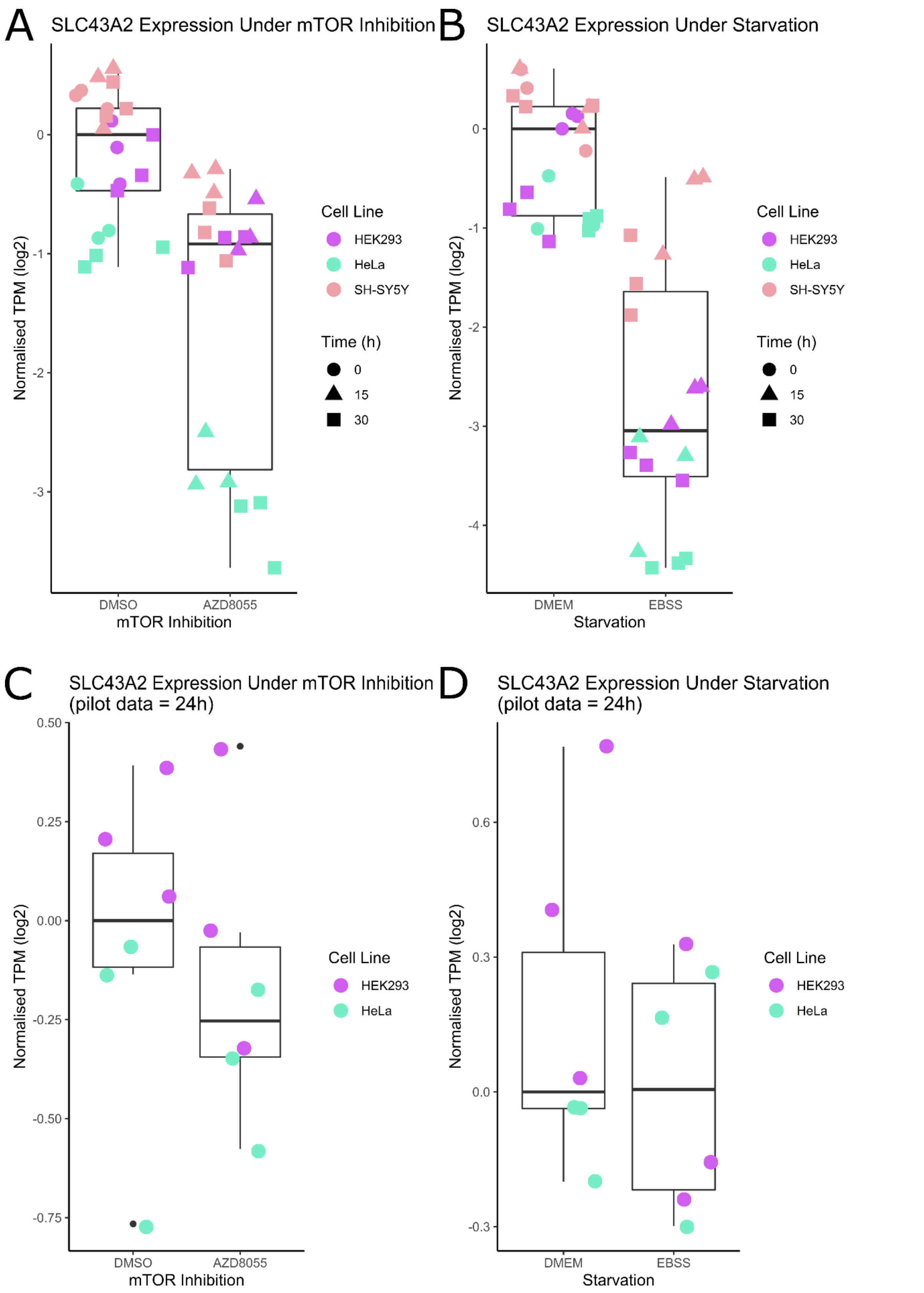

Supplementary Figure S7. Normalised SLC43A2 expression as transcripts per million (TPM) in primary experiments (A and B) and pilot experiments (C and D). mTOR inhibition and starvation experiments are represented in (A and C) and (B and D) respectively. Pilot experiments lacked SH-SY5Y data and were exposed to experimental stimulus for 24 hours.

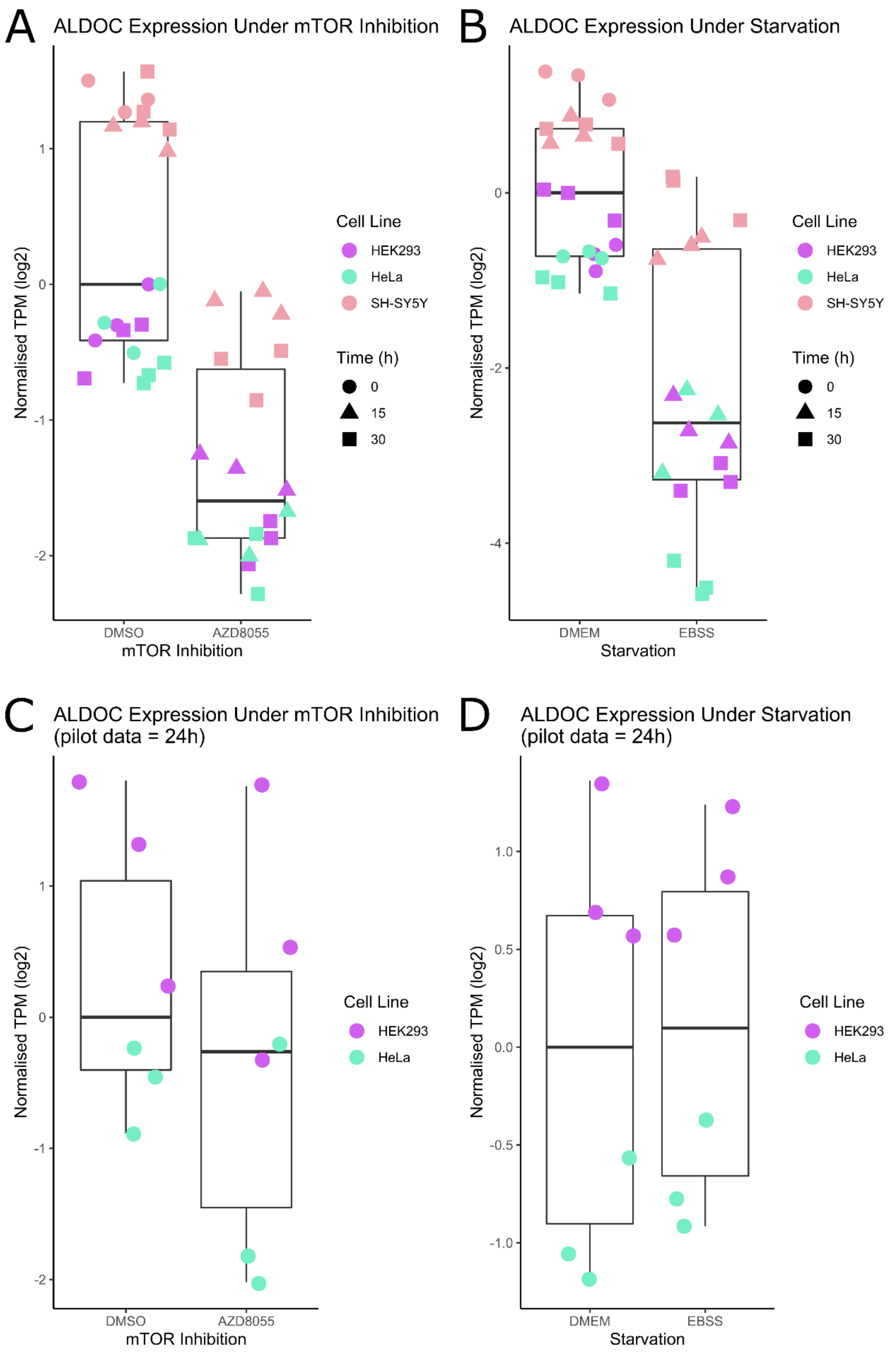

Supplementary Figure S8. Normalised ALDOC expression as transcripts per million (TPM) in primary experiments (A and B) and pilot experiments (C and D). mTOR inhibition and starvation experiments are represented in (A and C) and (B and D) respectively. Pilot experiments lacked SH-SY5Y data and were exposed to experimental stimulus for 24 hours.

Supplementary Figures S9 – S12: Genes discussed in manuscript that were significantly differentially expressed in the same direction for all cell lines in starvation experiments only

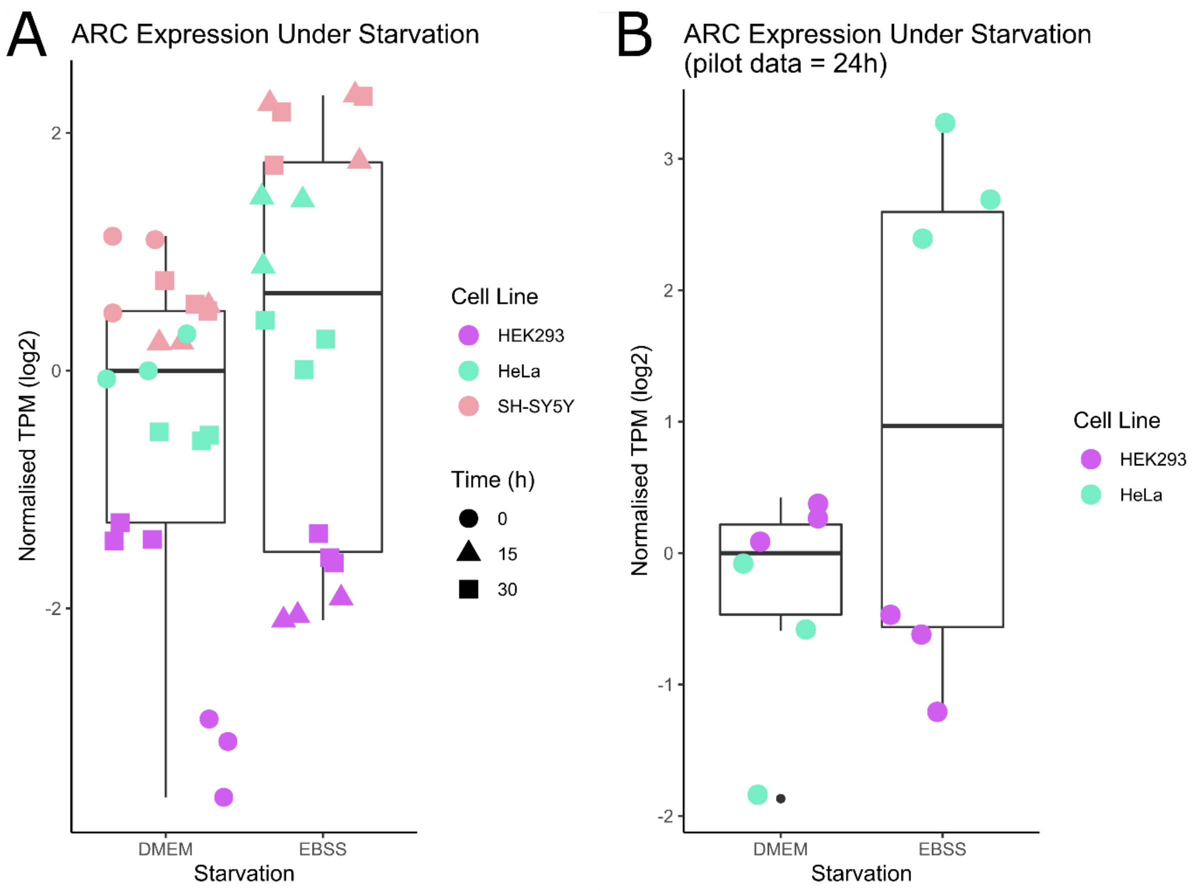

Supplementary Figure S9. Normalised ARC expression as transcripts per million (TPM) in primary experiments (A) and pilot experiments (B). Pilot experiments lacked SH-SY5Y data and were exposed to experimental stimulus for 24 hours.

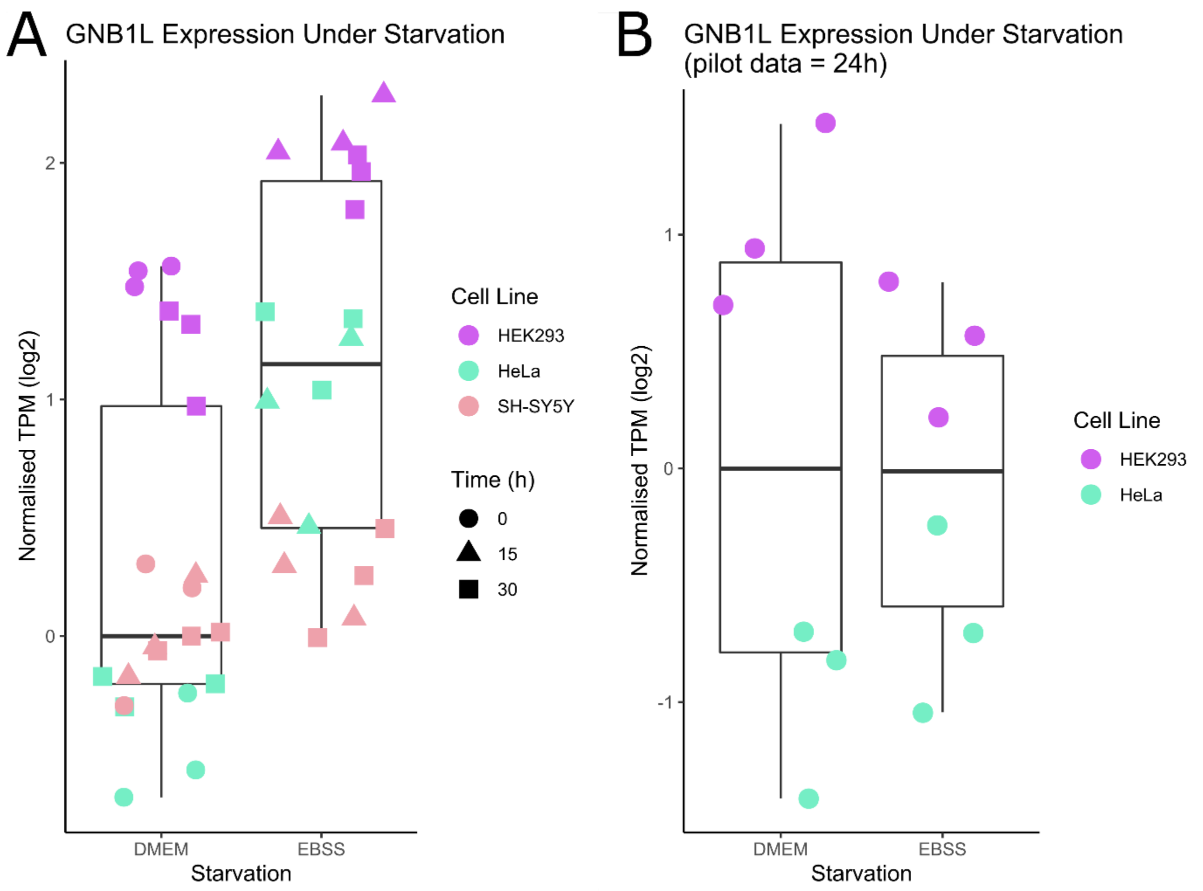

Supplementary Figure S10. Normalised GNB1L expression as transcripts per million (TPM) in primary experiments (A) and pilot experiments (B). Pilot experiments lacked SH-SY5Y data and were exposed to experimental stimulus for 24 hours.

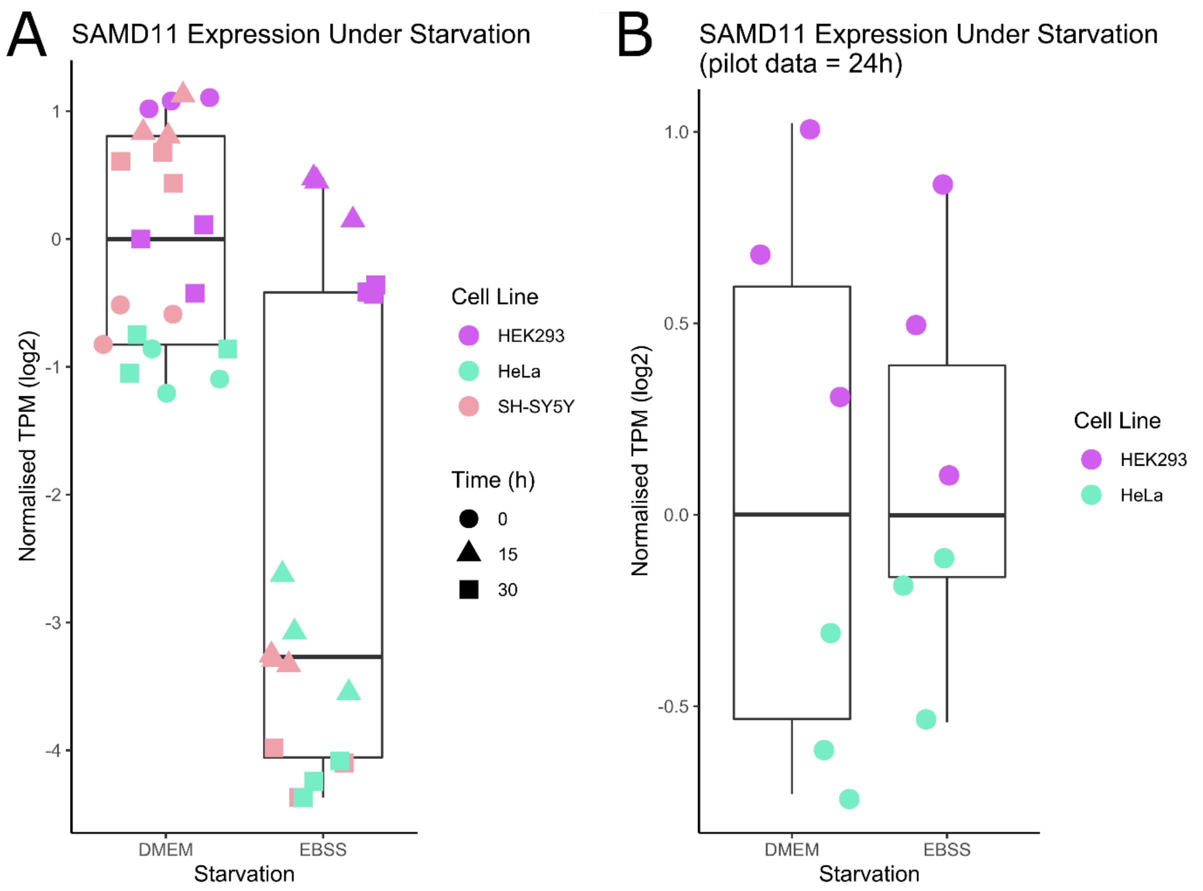

Supplementary Figure S11. Normalised SAMD11 expression as transcripts per million (TPM) in primary experiments (A) and pilot experiments (B). Pilot experiments lacked SH-SY5Y data and were exposed to experimental stimulus for 24 hours.

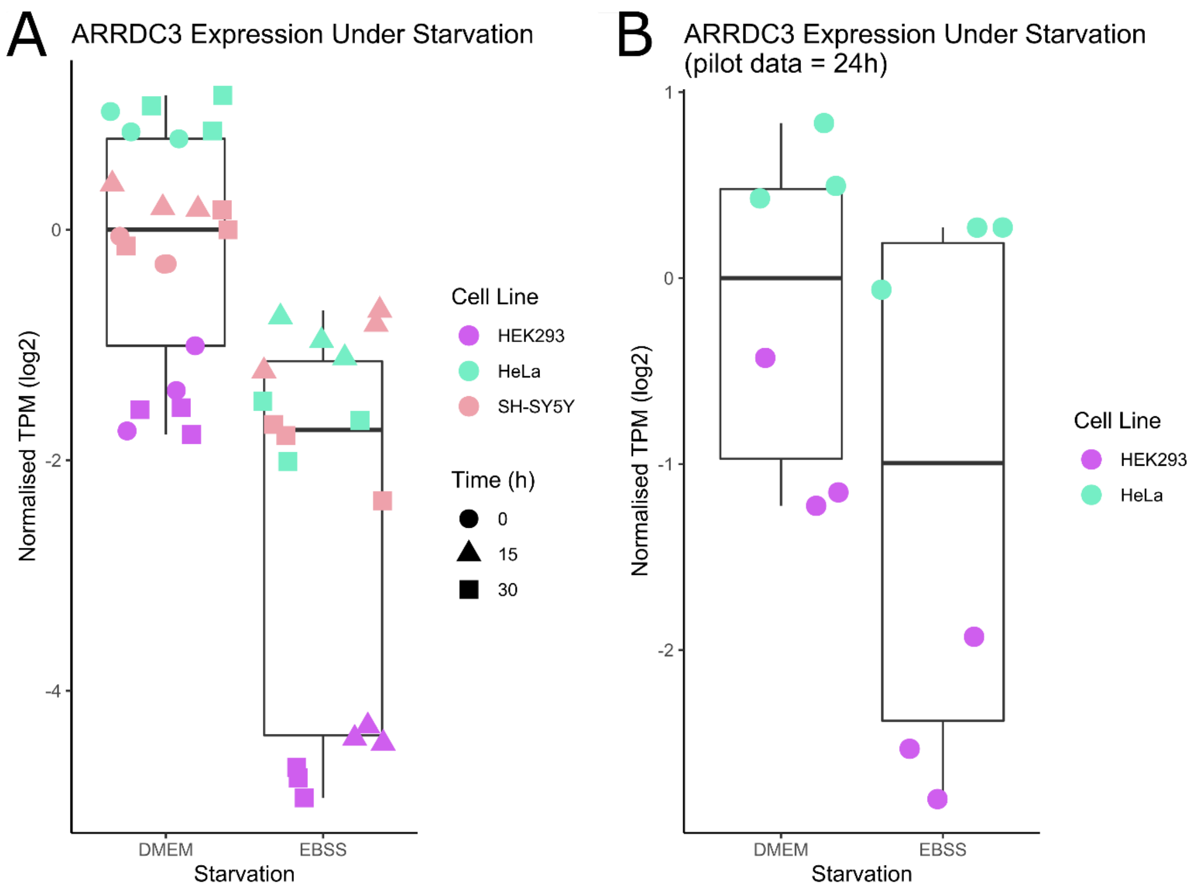

Supplementary Figure S12. Normalised ARRDC3 expression as transcripts per million (TPM) in primary experiments (A) and pilot experiments (B). Pilot experiments lacked SH-SY5Y data and were exposed to experimental stimulus for 24 hours.

Supplementary Figures S13 – S15: Genes discussed in manuscript that were significantly differentially expressed in the same direction for all cell lines in mTOR inhibition experiments only

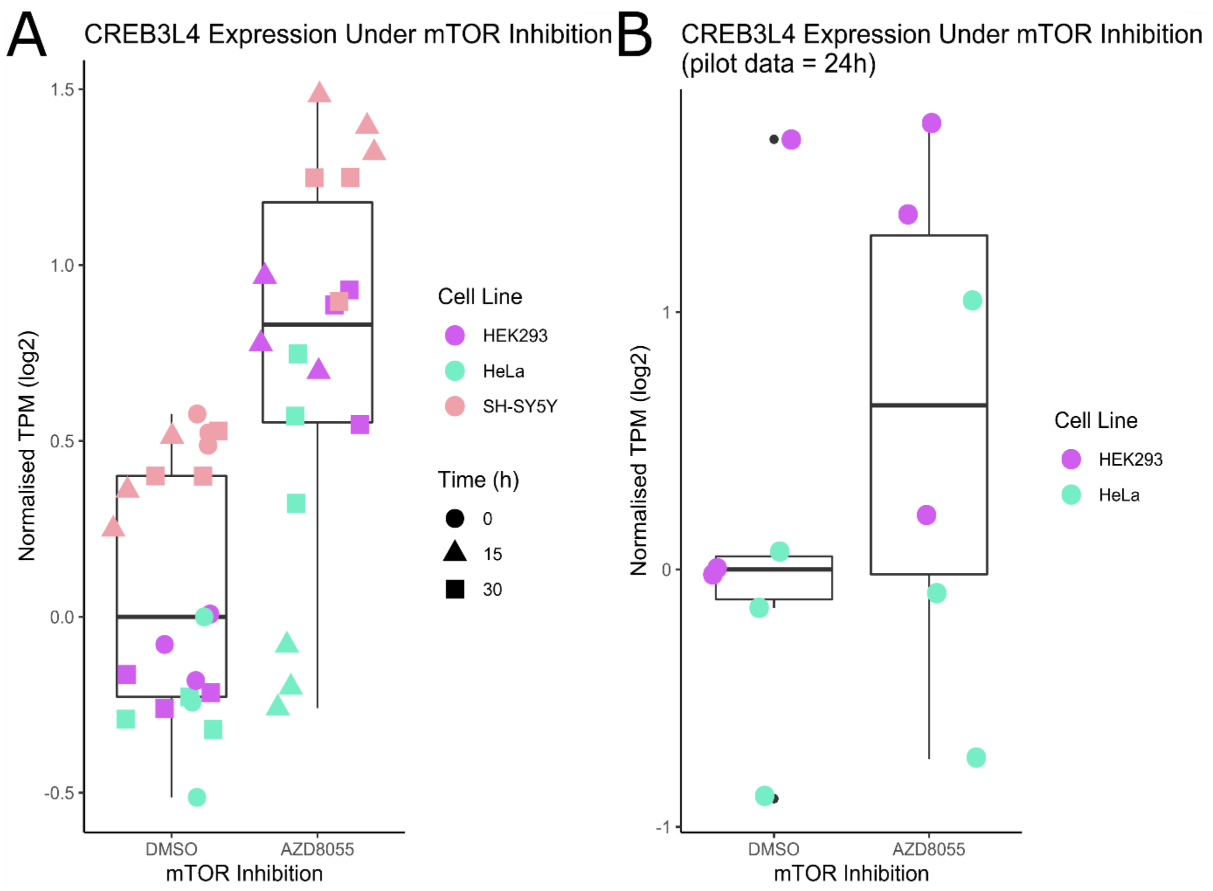

Supplementary Figure S13. Normalised CREB3L4 expression as transcripts per million (TPM) in primary experiments (A) and pilot experiments (B). Pilot experiments lacked SH-SY5Y data and were exposed to experimental stimulus for 24 hours.

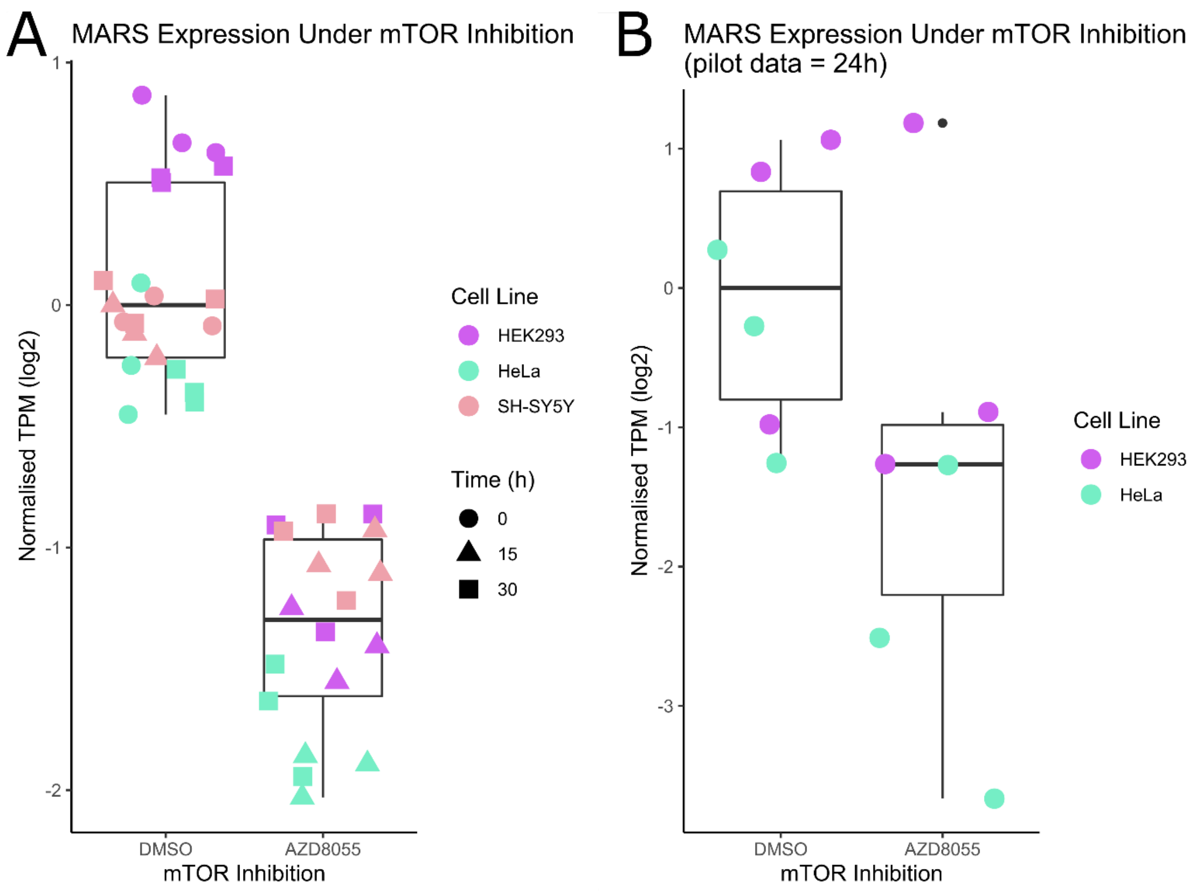

Supplementary Figure S14. Normalised MARS expression as transcripts per million (TPM) in primary experiments (A) and pilot experiments (B). Pilot experiments lacked SH-SY5Y data and were exposed to experimental stimulus for 24 hours.

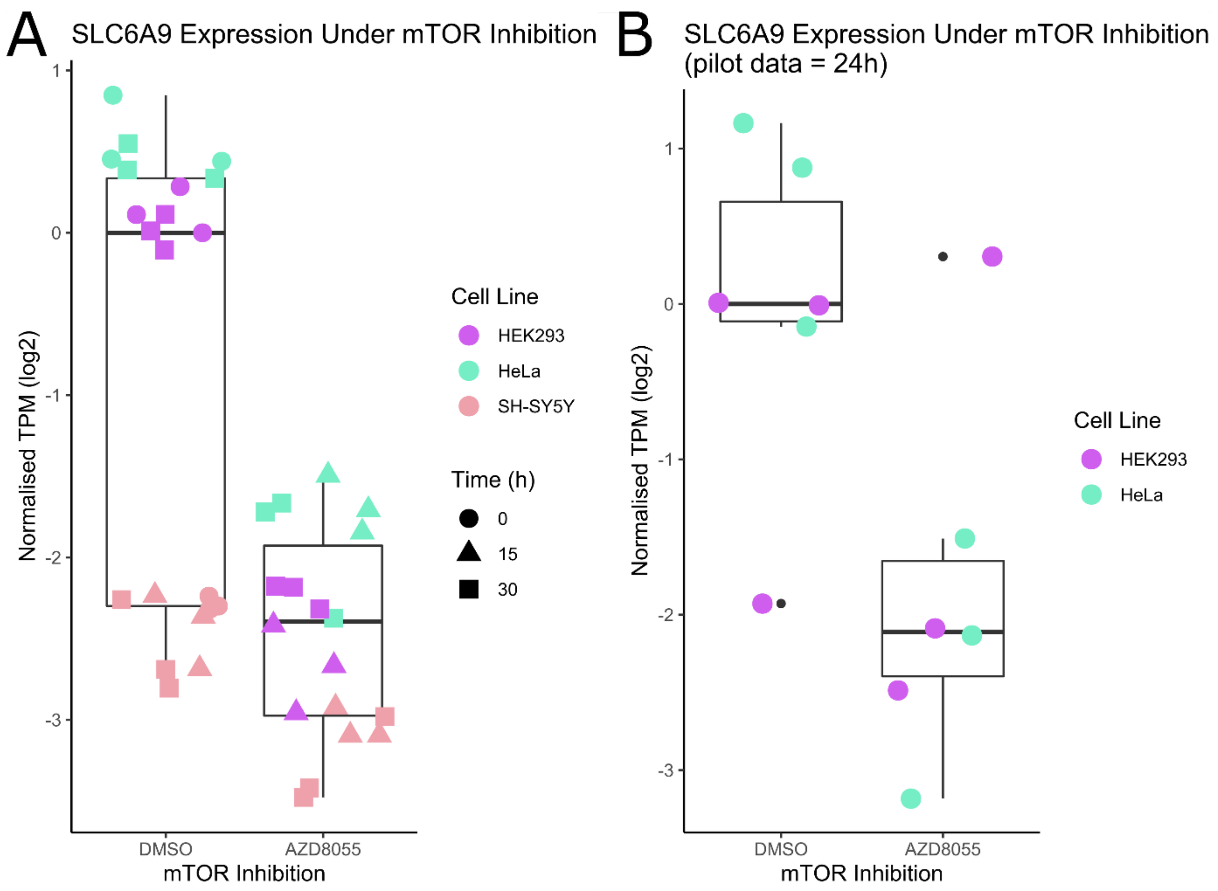

Supplementary Figure S15. Normalised SLC6A9 expression as transcripts per million (TPM) in primary experiments (A) and pilot experiments (B). Pilot experiments lacked SH-SY5Y data and were exposed to experimental stimulus for 24 hours.

Supplementary Figures S16 – S18: Genes discussed in manuscript that were significantly differentially expressed in the same direction for all cell lines in SH-SY5Y experiments only

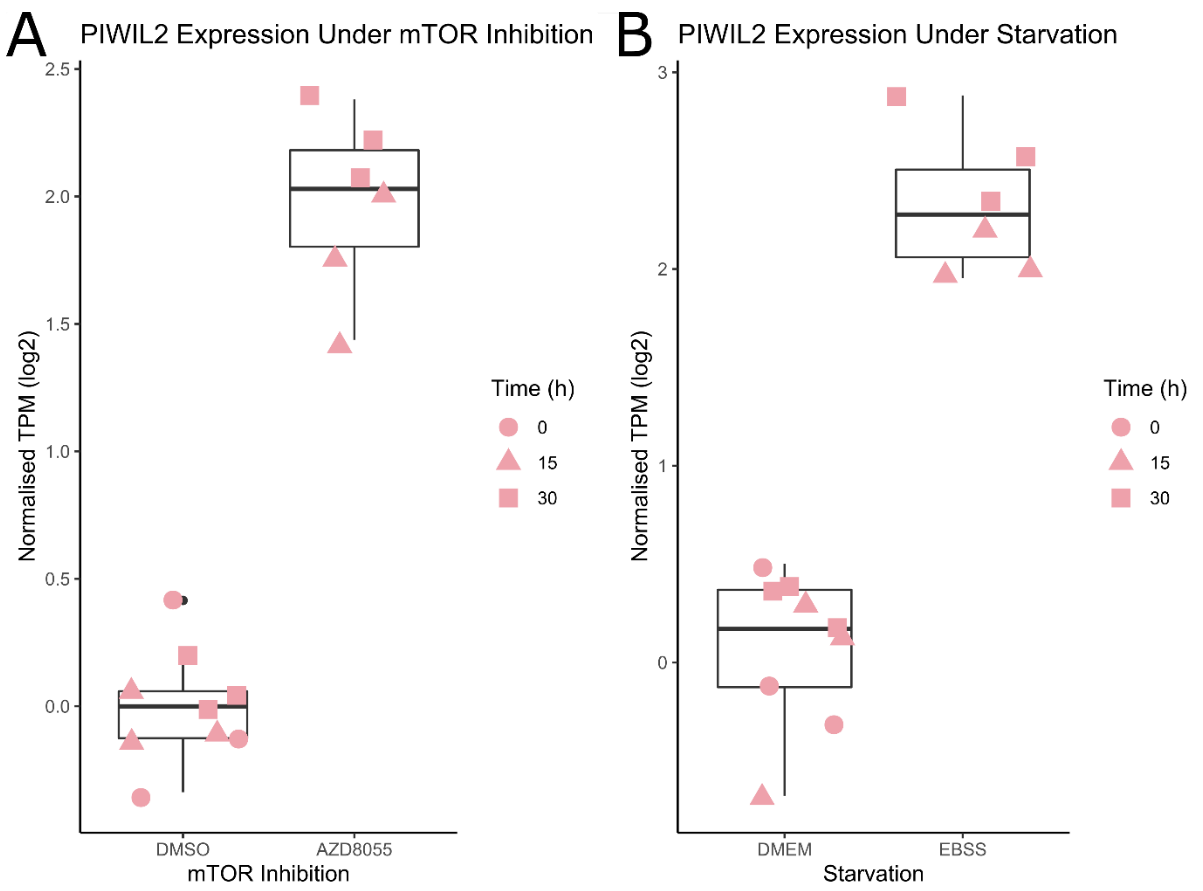

Supplementary Figure S16. Normalised PIWIL2 expression as transcripts per million (TPM) in primary experiments in SH-SY5Y cells for mTOR inhibition (A) and starvation (B) experiments

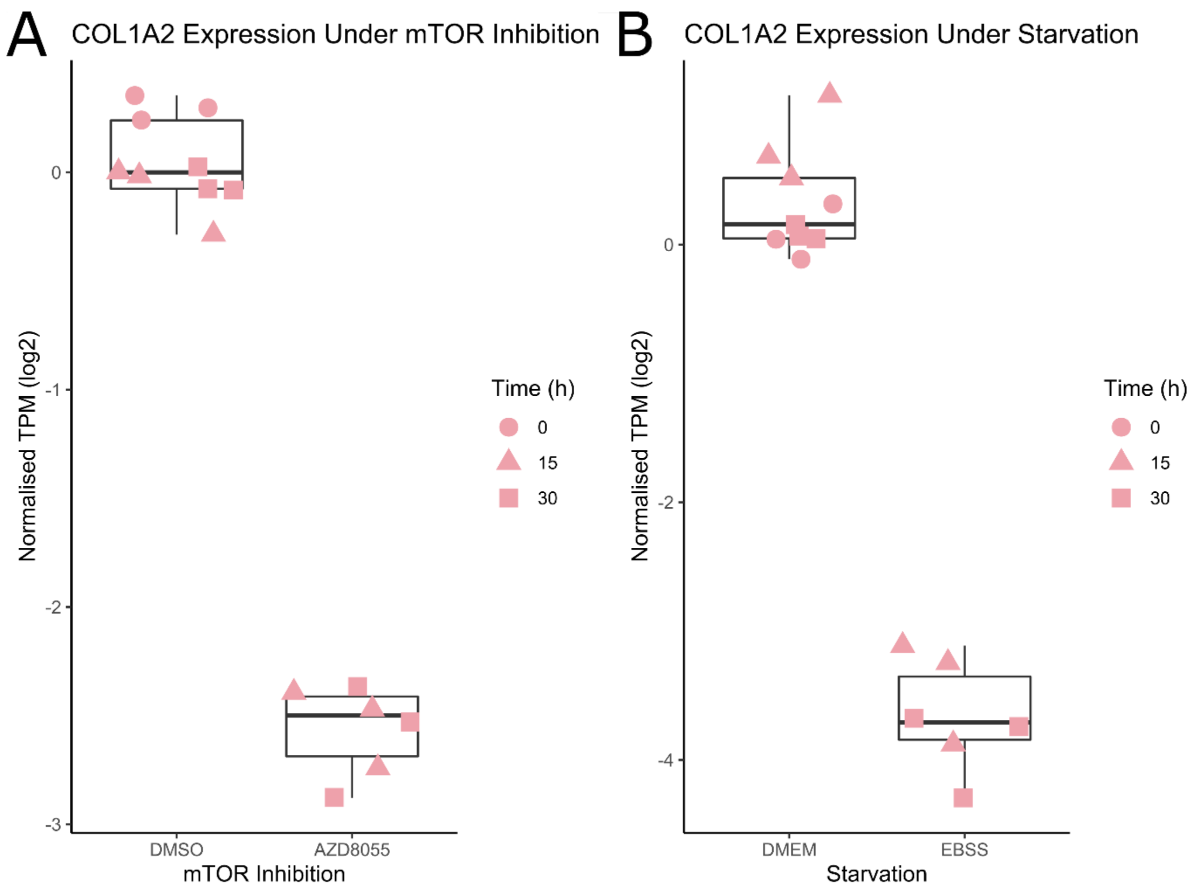

Supplementary Figure S17. Normalised COL1A2 expression as transcripts per million (TPM) in primary experiments in SH-SY5Y cells for mTOR inhibition (A) and starvation (B) experiments

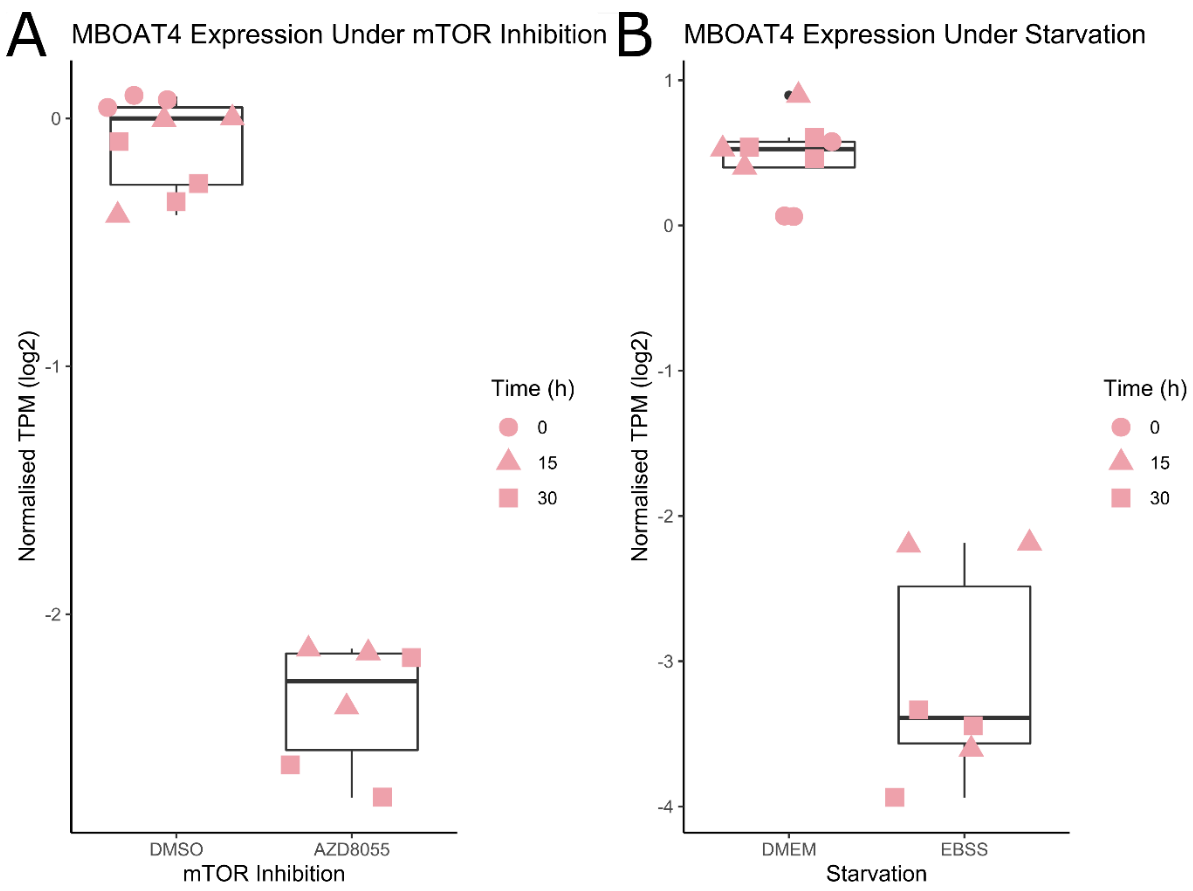

Supplementary Figure S18. Normalised MBOAT4 expression as transcripts per million (TPM) in primary experiments in SH-SY5Y cells for mTOR inhibition (A) and starvation (B) experiments

Supplementary Tables

Supplement tables S1-S6 (Supplement_tables_S1-S6.xlsx). Differential expression scores for the 6 experiments (3 cell lines x 2 conditions).

Supplement tables S7-S12 (supplement_tables_S7-S12.xlsx). Over-representation analysis of KEGG pathways enriched for DE genes - calculated for each of the 6 experiments (3 cell lines x 2 conditions).

Supplement tables S13-S18 (supplement_tables_S13-S18.xlsx). Signalling pathway impact analysis results - calculated for each of the 6 experiments (3 cell lines x 2 conditions).

Supplement tables S19-S24 (supplement_tables_S19-S24.xlsx). Over-representation analysis of transcription factor target gene sets that were specifically enriched for DE genes in each of the 6 experiments (3 cell lines x 2 conditions).

Supplement tables S25-S30 (supplement_tables_S25-S30.xlsx). Perturbation scores were calculated for the shared DE genes between a KEGG pathway and a TFT set for each of the 6 experiments (3 cell lines x 2 conditions).
